# Developmental Auditory and Speech–Language Performance in Pediatric Cochlear Implantation Recipients with Stable White Matter Lesions

**DOI:** 10.3390/brainsci13111540

**Published:** 2023-11-01

**Authors:** Huiru Fan, Dan Li, Wen Xie, Jing Wang, Huamao Cheng, Weijia Kong

**Affiliations:** 1 Department of Otorhinolaryngology, Union Hospital, Tongji Medical College, Huazhong University of Science and Technology, Wuhan 430022, China; 2 Department of Radiology, Union Hospital, Tongji Medical College, Huazhong University of Science and Technology, Wuhan 430022, China; 3 Hubei Province Key Laboratory of Molecular Imaging, Wuhan 430022, China; 4 Institute of Otorhinolaryngology, Union Hospital, Tongji Medical College, Huazhong University of Science and Technology, Wuhan 430022, China

**Keywords:** cochlear implantation, pediatric, white matter lesions, speech recognition, CAP, SIR

## Abstract

To analyze the association between stable asymptomatic white matter lesions (WMLs) and the cochlear implantation (CI) effect in congenitally deaf children, 43 CI children with stable asymptomatic WMLs determined via preoperative assessments and 86 peers with normal white matter were included. Outcome measurements included closed-set Mandarin Chinese (tone, disyllable, and sentence) recognition tests; categories of auditory performance (CAPs); and speech intelligibility rating (SIR) scales at 1, 12, and 24 months post-CI. Generalized estimating equation (GEE) models were used to analyze the association between WML and outcomes. In the WML group (control group), median CAP and SIR scores were 5 (5) and 4 (4) with mean rates of tone, disyllable, and sentence recognition of 84.8% (89.0%), 87.9% (89.7%), and 85.8% (88.0%) at 24 months post-CI, respectively. Auditory and speech performance improved significantly with implant use. Compared to their peers in the control group, for the participants with stable asymptomatic WMLs, auditory and speech abilities were not significantly different (*p* > 0.05). Stable asymptomatic WMLs might not be associated with poor auditory and speech intelligibility post-CI, which indicates that it is feasible to use comprehensive assessments to screen suitable candidates with WMLs who are likely to present with a good prognosis.

## 1. Introduction

Cochlear implantation (CI) is the most effective treatment for patients with congenital binaural severe-to-profound sensorineural hearing loss (SNHL) [1]. Numerous preoperative tests are performed to evaluate suitable candidates for surgery, including audiometry, auditory speech evaluation, and radiological assessments. Significantly, the effect of CI depends on central auditory pathway integrity [2]. The magnetic resonance imaging (MRI) of the head and inner ear has been routinely performed to evaluate inner ear malformations, cochlear nerve defects, and other brain structure abnormalities. A study including 157 patients with SNHL found 26 brain abnormalities in the MRIs for 23 patients with the most common abnormality being pure white matter changes in 13 patients [3]. Hong et al. reported that abnormal preoperative MRI with varying degrees of white matter changes was noted in 10 of 57 pediatric CI patients [4]. Jonas et al. found MRI brain abnormalities in 49 of 162 CI candidates, of which the most common were white matter changes [5]. Therefore, white matter lesions (WMLs) have been found to be common among brain MRI results in SNHL and CI children in previous studies.

CI outcomes in patients with WMLs have attracted researchers’ attention, as white matter is critical for transmitting action potentials between neurons. While improvements in communication abilities post-CI have been satisfactory in children [6,7,8], few studies have identified CI candidates with a potentially good prognosis, and the CI effect in children with WMLs remains variable [9,10,11,12,13]. Moon et al. reported that pediatric CI candidates with diffuse brain parenchymal lesions should be counseled regarding the poor prognosis preoperatively [9]. Chen et al. reported that children with WMLs had lower categories of auditory performance (CAPs) and speech intelligibility rating (SIR) scores than the control without WMLs at 24, 48, and 60 months post-CI [10]. Similarly, Zhao et al. showed significant differences in semantic auditory behavior ability and expressive language skills between children with and without WMLs 24 months after CI [11]. However, Wang et al. observed that children with WMLs confirmed by two MRI scans obtained better CAP and SIR scores with 36 months of implant use [12]. In addition, Shen et al. reported no significant difference in the CAP and SIR scores of children with WMLs and normal white matter two years post-CI rehabilitation [13]. With the increasing number of SNHL children, more children with WMLs are potential CI candidates. Among the children with SNHL with WMLs, the identification of CI candidates who have the potential for good habilitation outcomes deserves further evaluation.

If WMLs are detected on MRI, many probable white matter disorders are suspected, both acquired and inherited, for which the diagnostic process is slow and difficult in the early stages of the disease [14,15]. In pediatric patients, inherited white matter disorders—leukodystrophies—deserve much attention. However, the diagnosis of leukodystrophies is expensive and challenging in most cases, as clinical manifestations can present at any age [15,16]. It is recommended that the clinical signs and symptoms be classified as neurological manifestations, including motor impairment, dysautonomia, cognitive impairment, psychiatric disorders, seizures, peripheral nerve involvement as well as other neurological manifestations; and non-neurological manifestations [16]. The first clinical manifestations of leukodystrophies are often nonspecific and can occur in different ages from neonatal to late adulthood [16]. In general, age of onset typically correlates inversely with disease severity and rate of progression [15]. Given the much poorer prognosis of early-onset and progressive leukodystrophies, the outcomes of CI for children who are suspected of such diseases would not be satisfactory. For children with some WMLs with none or delayed-onset symptoms and a good prognosis from WMLs, benefits in auditory and language development from CI are expected during early childhood, which can significantly improve their quality of life. Therefore, identifying CI candidates with WMLs that are likely to exhibit a good prognosis by evaluating their habilitation outcomes are issues that deserve attention.

This study aimed to (1) evaluate auditory and speech–language performance in pediatric CI recipients with stable asymptomatic WMLs and (2) analyze the association between stable asymptomatic WMLs and the rehabilitation effect.

## 2. Materials and Methods

### 2.1. Participants

Participants were pediatric CI recipients in the Department of Otorhinolaryngology and Head and Neck Surgery, Wuhan Union Hospital, between January 2013 and September 2018. This prospective study recruited 129 participants, of which 43 presented with stable asymptomatic WMLs and 86 presented with normal white matter (control). The inclusion criteria were as follows: (1) children were determined to have congenital bilateral severe to profound SNHL using a combination of subjective and objective audiological assessments preoperatively and received unilateral CI before 18 years of age; (2) children had experience of wearing a hearing aid (HA) for at least 3 months pre-CI; (3) stable asymptomatic WMLs or normal white matter (control) were determined via preoperative evaluation; (4) children were enrolled in a rehabilitative program for at least 3 months post-CI. The exclusion criteria were as follows: (1) children with other abnormalities on MRI or other systemic diseases; (2) children with major operative or postoperative complications; (3) children who accepted a second CI during the follow-up period; (4) children with auditory neuropathy spectrum disorder were excluded from this study, but some children who were determined to have an OTOF1 mutation were still included. Stable asymptomatic WMLs were defined as WMLs that did not progress between two serial MRI scans six months apart in children that had normal results in metabolic screening and systematic evaluations, including growth, intelligence, cognitive, and motor function development, by pediatric neurology department experts. Metabolic screening was performed to rule out common aliphatic acid, organic acid, and amino acid metabolism disorders. The demographic information and clinical materials of all participants were collected, including gender, parental literacy, residential setting, preoperative audiological assessments and experience of wearing HA, auditory and speech rehabilitation training experience and Mandarin Meaningful Auditory Integration Scale (MMAIS)/Infant–Toddler (IT-MMAIS) score pre-CI, age at implantation, implanted ear, CI type, and mean hours of implant use per day. This research was approved by the Ethics Committee of Wuhan Union Hospital.

### 2.2. WML Evaluation: Fazekas Score

**Figure 1:**
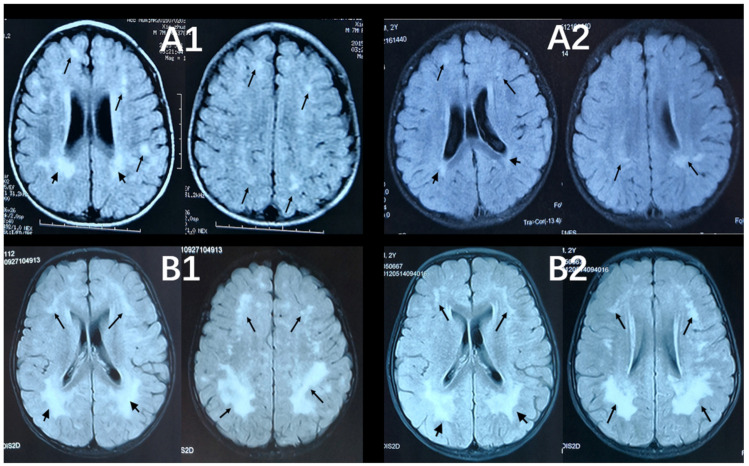
Axial, T2-FlAIR cerebral MRI scans six months apart of the individuals. Short black arrows point to lesions in periventricular white matter. Black arrows point to lesions in deep white matter. (**A1**) The first MRI scan for a 18-month-old boy showed extensive WMLs (PVH = 3, DWMH = 2, Fazekas score = 5). (**A2**) The second scan for the same child (24 months of age) showed improvement in WML with the Fazekas score declining (PVH = 2, DWMH = 2, Fazekas score = 4). (**B1**,**B2**) Two MRI scans of a 31-month-old (date of the second MRI scan) boy showed no obvious change in diffuse WMLs (PVH = 3, DWMH = 3, Fazekas score = 6).

T1-weighted (T1-WI), T2-weighted (T2-WI), and T2-fluid attenuated inversion recovery sequence (T2-FLAIR) images were available for participants and interpreted by an experienced neuroradiologist. The Fazekas scale was used to assess the degree of periventricular hyperintense (PVH) and deep white matter hyperintense (DWMH) (Table 1) [17]. The total Fazekas score is the sum of the PVH and DWMH scores. Two Fazekas scores based on their two MRI scans were consistent in 42 children, and one child’s scores declined. Their second Fazekas score was used in the statistical analysis. Figure 1 shows T2-FLAIR images of two cases with annotated Fazekas scores six months apart, including the child whose score declined.

### 2.3. CI Outcome Evaluation: Auditory and Speech–Language Abilities

Objective and subjective scale assessments were both used to evaluate rehabilitation outcomes post-CI. Auditory outcomes were assessed using categories of auditory performance (CAP) scale [18] and speech recognition tests [19], including closed-set Mandarin Chinese tone, disyllable, and short sentence recognition. Speech–language ability was evaluated using the speech intelligibility rating (SIR) scale [18]. Children’s main caregivers assisted with CAP and SIR scales (categorizing criteria are described in Supplemental Tables S1 and S2) evaluation, of which the reliability was confirmed [20,21]. The speech recognition test was performed on both ears simultaneously (with the opposite ear unaided). All assessments were performed by appointed experienced audiologists in the rehabilitation institutes at the following time points: 1, 12, and 24 months after device activation.

### 2.4. Statistical Analysis

Statistical analyses were performed using SPSS software (version 25, IBM SPSS, Armonk, NY, USA). Values were given as absolute frequency distribution, mean (standard deviation, SD), and median (interquartile range). The association between WML and outcomes as well as the interaction between WML and time after device activation were analyzed using a generalized estimating equation (GEE), including unadjusted and adjusted models (working correlation matrix structure: unstructured correlation). Model 1 was an unadjusted model. Model 2 aimed to analyze the interaction between WML and time. Model 3 controlled for time after device activation, age at implantation, parental literacy, residual hearing, rehabilitation experience, and MMAIS/IT-MMAIS score pre-CI. Linear regression models were established for speech recognition rates. For CAP and SIR scores, dichotomous logistic regression models were established by converting dependent variables to dichotomous variables based on the half-cut values (CAP scores of ≥4 and <4; SIR scores of ≥3 and <3). A possible association between Fazekas score and CI outcomes was further analyzed. The results are presented as point estimates and 95% confidence intervals (95% CI) for the regression coefficients and odds ratios (ORs). To examine the robustness of the estimation, we performed a post hoc sensitivity analysis by restricting subjects to those who received CI before the seventh year of life. Spearman’s rank correlation and Mann–Whitney *U* test were used in the exploratory analyses. The significance level was set to *p* < 0.05.

## 3. Results

### 3.1. Clinical Characteristics and WML Imaging Assessment

Table 2 shows 129 participants’ detailed demographic information and clinical characteristics. The study included 73 (56.6%) boys, and the age at CI ranged from 1.7 to 17.5 years (median 3.5 years, quartiles 2.8–5.4 years). Senior middle school and above accounted for 38.0% of the parental literacy levels. Regarding preoperative audiological assessments, pure tone audiometry thresholds at 0.5, 1, 2, and 4 kHz could not be recorded up to 120 dB nHL in 65 (50.4%) of the participants. Before surgery, 51.2% children had experience of wearing an HA for more than 12 months, and 69.8% children had undergone auditory–verbal rehabilitation training for more than 3 months. The device implanted was a Nucleus in 78 patients, MED-EL in 33 patients, and Advanced Bionics in 18 patients. Electrode insertion was complete, as responses from all electrode arrays were successfully recorded after activation.

**Figure 2:**
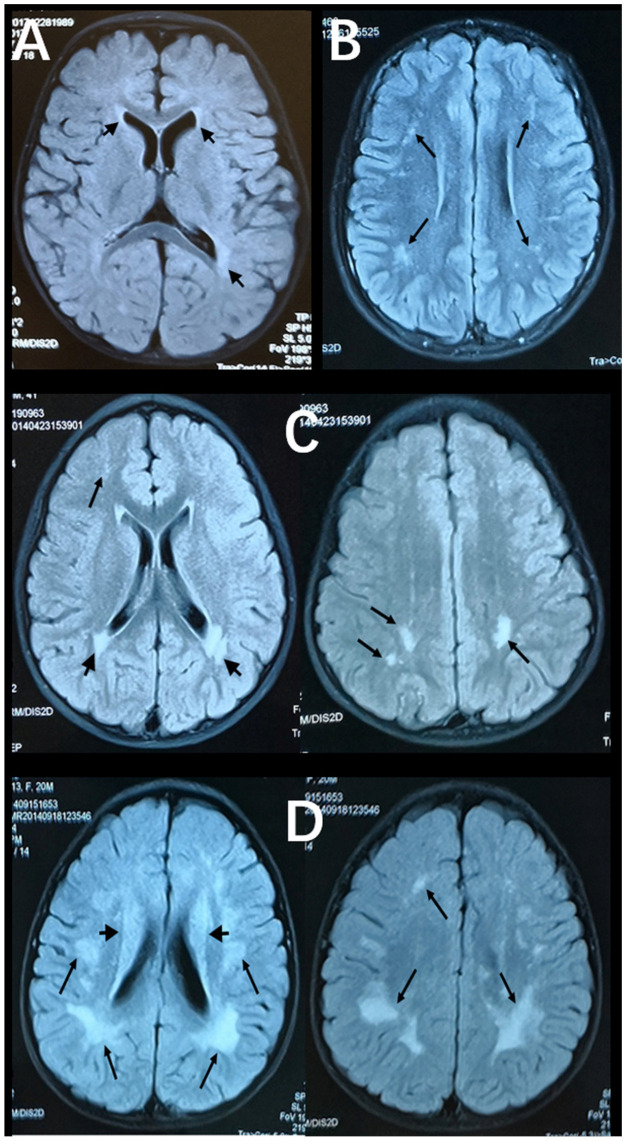
Axial, T2-FlAIR cerebral MRI scans with annotated Fazekas scores. (**A**) Multifocal WMLs in periventricular white matter (short black arrows, PVH = 1, Fazekas score = 1). (**B**) Multifocal WMLs in deep white matter (black arrows) of the frontal and parietal lobes (DWMH = 2, Fazekas score = 2). (**C**) Extensive WMLs in periventricular white matter (short black arrows) and deep white matter (black arrows) of the frontal and parietal lobes (PVH = 3, DWMH = 2, Fazekas score = 5). (**D**) Diffuse WMLs in periventricular white matter (short black arrows) and deep white matter (black arrows) of whole brain areas (PVH = 2, DWMH = 3, Fazekas score = 5).

**Figure 3:**
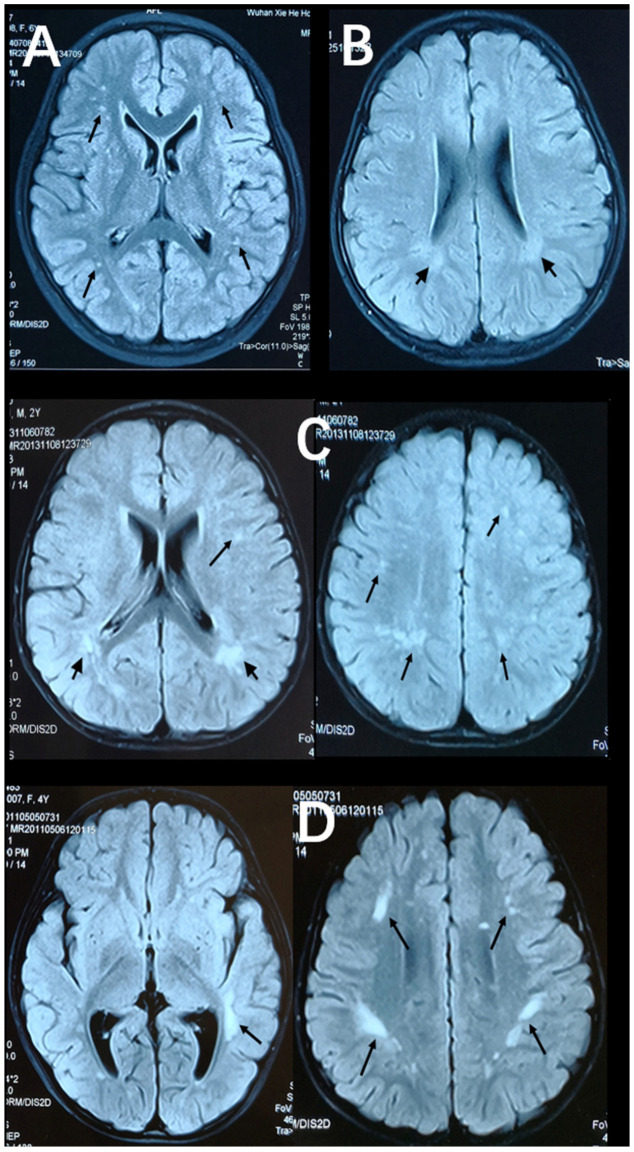
Axial, T2-FlAIR cerebral MRI scans with annotated involved areas. (**A**) The frontal and occipital lobes involvement (black arrows). (**B**) Periventricular white matter involvement (short black arrows). (**C**) The frontal and parietal lobes involvement (black arrows) together with periventricular white matter involvement (short black arrows). (**D**) The temporal, frontal and parietal lobes involvement (black arrows).

Figure 2 and Figure 3 show representative T2-FLAIR images with annotated Fazekas scores and involved areas. The numbers of participants with Fazekas scores of 1, 2, 3, 4, 5, and 6 were 15, 15, 2, 5, 4, and 2, respectively. PVH was detected in 25 participants and DWMH was detected in 32 participants. PVH and DWMH were detected concurrently in 14 participants. The frontal lobe (31/32) was the most frequently involved area of deep white matter, which was followed by the parietal lobe (22/32) and the occipital lobe (11/32). The temporal lobe (10/32) was the least involved area. Table 3 shows the distributions of Fazekas scores and involved areas.

### 3.2. Post-CI Rehabilitation Outcomes

All the participants reported longer than 8 h of implant use per day. School-age children of participants attended ordinary classes in mainstream primary schools at the last follow-up meeting. In both groups, the participants’ speech recognition rates, CAP and SIR scores improved gradually over time and increased rapidly during the first year post-CI (Table 4). Participants in the WML group reported no clinical symptoms, such as motor disfunction or cognition impairment, etc., during the follow-up period post-CI. In the WML group, the mean (SD) tone, disyllable, and short sentence recognition rates were 23.5% (31.8%), 21.7% (27.0%), and 13.2% (18.3%) one month after device activation, respectively. At 12 months post-CI, the three mean (SD) rates were 73.4% (28.1%), 77.7% (26.1%), and 75.2% (25.6%), respectively. Impressively, at 24 months post-CI, their three mean (SD) rates were 84.8% (23.8%), 87.9% (20.9%), and 85.8% (20.8%), respectively. The median CAP score was 1 one month post-CI, 4 twelve months post-CI, and 5 twenty-four months post-CI. Their median SIR score was 1 one month post-CI, 3 twelve months post-CI, and 4 twenty-four post-CI. In the control group, the five outcome values showed a similar improvement trend over time.

### 3.3. Association Analysis between WML and CI Outcomes

**Figure 4:**
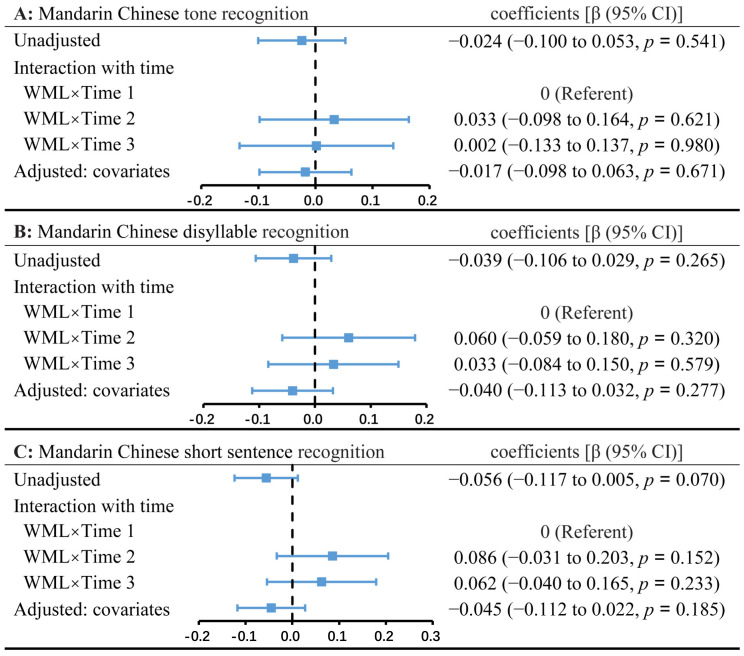
Association of WML with Mandarin Chinese tone (**A**), disyllable (**B**), and short sentence (**C**) recognition outcomes. The point estimate and 95% confidence interval (95% CI) for the coefficients with WML are presented for an unadjusted model (model 1), a model aiming to analyze the interaction between WML and time (model 2), and another model adjusted for covariates (model 3). The independent variables in model 2 were WML, time after device activation, and the interaction between WML and time after device activation. Model 3 was adjusted for time, age at implantation, parental literacy, residual hearing, experience of rehabilitation pre-CI, and MMAIS/IT-MMAIS score pre-CI.

**Figure 5:**
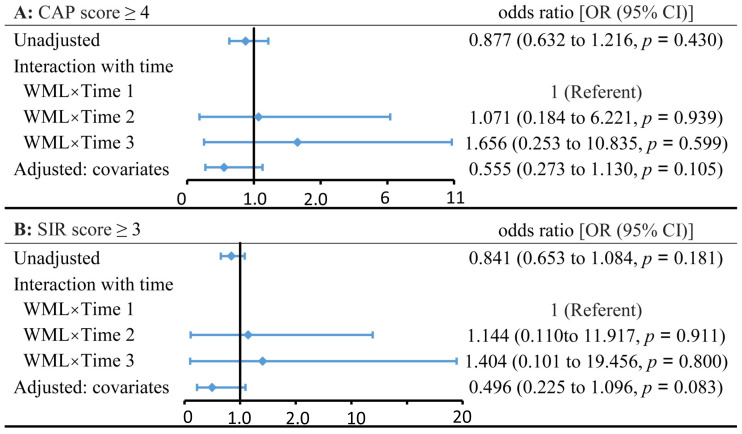
Association of WML with CAP (**A**) and SIR (**B**) outcomes. The point estimate and 95% confidence interval (95% CI) for the odds ratios (ORs) with WML are presented for an unadjusted model (model 1), a model aiming to analyze the interaction between WML and time (model 2), and another model adjusted for covariates (model 3). The independent variables in model 2 were WML, time after device activation, and the interaction between WML and time after device activation. Model 3 was adjusted for time, age at implantation, parental literacy, residual hearing, experience of rehabilitation pre-CI, and MMAIS/IT-MMAIS score pre-CI.

Unadjusted (model 1), interaction (model 2), and adjusted (model 3) GEE models were established separately for the Mandarin Chinese tone (Figure 4A), disyllable (Figure 4B), and short sentence (Figure 4C) recognition. No significant associations were found between WML and Mandarin Chinese speech recognition in univariate and multivariate adjusted regression models (*p* values > 0.05, Figure 4). Regarding the CAP and SIR scores using the GEE dichotomous logistic regression models (Figure 5A,B), compared with the control, participants with WML had nonsignificant ORs of 0.555 (95% CI: 0.273–1.130; *p* = 0.105) for CAP scores ≥ 4 and 0.496 (95% CI: 0.225–1.096; *p* = 0.083) for SIR scores ≥ 3 in the adjusted models. No interaction between WMLs and time was found for CI outcomes (Figure 4 and Figure 5). In a post hoc sensitivity analysis of WML, we obtained similar results using adjusted models by restricting participants who received CI at younger than 7 years of age (*n* = 117, Table 5). Models were adjusted for time after device activation, age at implantation, parental literacy, residual hearing, rehabilitation experience, and MMAIS/IT-MMAIS score pre-CI. In the WML group, for tone, disyllable, and short sentence recognition rates, as well as CAP and SIR scores, the adjusted GEE models indicated that every value improved significantly over time (Table 6). Table 6 shows significant differences between different time points with a reference of 1 month after activation (each *p* < 0.001). Regarding the association between Fazekas score and CI outcomes, no significant odds ratios or coefficients were obtained in the adjusted models (Table 6).

In the exploratory analyses, it was found that Fazekas scores were not correlated with tone, disyllable, and short sentence recognition rates or CAP and SIR scores at 12 months post-CI. Similarly, no significant correlations were found at 24 months post-CI (Table 7). Because of the Fazekas score’s unbalanced distribution, the detailed outcomes of participants with Fazekas scores of 5 and 6 were described in Supplemental Table S3, which shows that auditory and speech abilities developed steadily in these participants post-CI. In Supplemental Table S4, the participants in subgroups with and without temporal lobe involvement showed improved speech recognition rates, CAP scores, and SIR scores at 24 months post-CI. Furthermore, the Mann–Whitney U test found no significant difference between the subgroups with and without temporal lobe involvement.

## 4. Discussion

This study observed CI outcomes in children with stable asymptomatic WMLs and with normal white matter. Using both objective tests and subjective scales, our study demonstrated that children with stable asymptomatic WMLs gained satisfactory hearing and speech abilities post-CI. The Mandarin Chinese speech recognition, CAP, and SIR scores of participants with stable asymptomatic WMLs developed significantly in the first two years post-CI. The CAP and SIR scale assessment results were also consistent with the results in children with WML reported by Chen et al. and Shen et al. [10,13]. In addition, the auditory and speech development tendencies of participants in the WML group were similar to those of the control group. Wang et al. observed that children with WMLs obtained better CAP and SIR scores after at least 36 months of implant use [12]. Many auditory and language rehabilitation professionals reported that performance in the early post-CI stage was critical to long-term CI effect [22,23,24]. Given the significant development of hearing and speech-language abilities of participants with stable asymptomatic WMLs in the first two years post-CI, we could expect that the longitudinal CI effects in these children would be good.

In the adjusted GEE models, no significant associations were found between WML and CI outcomes together with an interaction between WML and time. While many studies have reported that children with WMLs gained some benefits from CI, whether any differences existed between the outcomes in children with WML and normal white matter remains inconsistent. Chen et al. reported that while CAP and SIR scores in children with WMLs improved over time, they were still poorer than the results of the control group at 24 months post-CI [10]. Wang et al. performed a cross-sectional observation study on CI outcomes in 40 children with WML in which the majority of participants obtained a satisfactory postoperative effect, but a weak correlation was reported between the degree of WML on brain MRI and long-term CAP and SIR scores [12]. However, Shen et al. reported no significant difference in IT-MAIS/MAIS scores between children with and without WMLs in the first year of implant use [25]. In another study, the authors reported no significant differences in CAP and SIR scores between children with and without WMLs at approximately two years post-CI [13]. Zhang et al. reported no significant difference in CAP scores but a marked difference in SIR scores between children with WMLs and their CI peers at 12 and 24 months post-CI [26]. It was noted that objective tests and subjective scale assessments were both used to evaluate CI outcomes in our study, while only subjective scale assessments were used in the above literature. The inconsistent results might be due to possible changes in WML during implant use or a severe degree of WML in the subgroup. Screening for metabolic abnormalities was not mentioned in these studies except in the Shen et al. study [13]. The patients in the study by Chen et al. [10] did not receive second MRI scans, which were performed in studies by Shen et al. [13,25] and Wang et al. [12]. In our study, stable asymptomatic WMLs were confirmed through a comprehensive preoperative evaluation strategy, including metabolic screening, two sets of MRI scans, neural system function evaluation, and six-month observation evaluation, which we further summarized later. It could be noted that participant screening methods differed among previous studies, potentially explaining some differences in these results.

Given that none of standard methods have been recommended to assess or screen CI candidates with WMLs in clinical practice and the literature until now, we try to summarize a feasible method to evaluate such candidates. Firstly, given the potential positive effect of young age at CI and myelin development process in children, two sets of MRI scans six months apart were performed to observe WML changes or progression [15]. Secondly, candidates’ development, cognitive function, and nervous system function were confirmed normal by pediatric experts. Thirdly, candidates with WMLs underwent metabolic screening and showed normal results. Finally, neither progression of WML on MRI nor clinical signs and symptoms occurred during the at least six-month waiting and observational period before surgery. After the comprehensive assessments, candidates with severe, rapidly progressive leukoencephalopathy and hereditary metabolic disorders related to amino and organic acid metabolism could be excluded. Moreover, no symptoms like motor impairment, dysautonomia, or cognitive impairment were observed in participants during their 24 months of follow-up observation post-CI, which might indirectly indicate that the WMLs in this study’s participants were stable and the methods were feasible to evaluate candidates for a potential good prognosis. In summary, the methods could be named “preoperative comprehensive evaluation strategy for CI candidates with WML (PCES-WML)”.

White matter comprises neuronal fibers and myelin. Myelination begins in utero and is largely complete on MR imaging by 24 months of life [27]. Although myelination develops rapidly in the neonatal period, this process can continue until adolescence [28]. It is a critical and difficult task to evaluate the WML when a radiologist faces MR imaging of the incompletely myelinated brain of a child, because many CI candidates are young children. Park et al. reported that the age-related development of white matter tracts might continue until eight years of age in deaf children and that cerebral white matter tract development was delayed in prelingually deaf children compared with typical hearing children [29]. Delayed myelin development might be one of the possible causes of WMLs in children with SNHL. Prominent WMLs with asymmetric and multifocal patterns in brain MRIs are mostly observed in acquired white matter abnormalities, while symmetrical forms usually suggest heritable white matter disorders [16,30]. Although some distinction features exist, presentation and imaging findings are usually mimicked in a variety of white matter disorders. Therefore, it is worth much attention to evaluate the prognosis and degree of WMLs detected on MRI in children with SNHL.

The WMLs’ degree can be evaluated with various rating systems. The Fazekas scale is a widely used measurement [31,32]. Moon et al. observed the progress in auditory and speech outcomes in children two years post-CI and found a significant difference between patients with brain lesions and the control without lesions (*n* = 27) [9]. The authors also reported that the diffuse cerebral WML groups (>10% of the whole white matter, *n* = 12) showed the worst results among all groups (focal WML, *n* = 7, extra-axial lesion, *n* = 3, ventriculomegaly, *n* = 5, and control group, *n* = 27) [9]. In addition, the patients in the brain lesion group presented additional disabilities in the study by Moon et al. [9]. Wang et al. reported a weak correlation between the degree of WML and CAP as well as SIR scores [12]. Chen et al. observed that Fazekas scores were negatively correlated with CAP and SIR scores [10]. Zhang et al. graded WML using the Scheltens scale and found that the auditory–speech performance was highly related to WML grading [26]. Our results were inconsistent with these reports. No association between the Fazekas score and CI outcomes was found in the adjusted models and exploratory correlation analysis. On the one hand, in order to remove the effect of potential confounding factors, such as age at CI, the factors were adjusted by including them in the models. On the other hand, Fazekas scores of 1 and 2, considered mild lesions, accounted for 69.8% of participants with WML in our study. In addition, lesions with Fazekas scores of 5 and 6, considered severe, were observed to be stable and asymptomatic during the study course. If the extent of WMLs is not severe enough to interrupt auditory perception and language production processing, then it may not be associated with poor outcomes.

The Fazekas scale divides WMLs into two sections for evaluation (PVH and DWMH) since WMLs of different degrees and locations show characteristic histopathologic changes [33]. The temporal lobe (10/43) was the least involved area in the study. Actually, increased focus on temporal lobe lesions was warranted due to the fact that the temporal lobe is a critical neural center of cognition and speech–auditory function. A four-year-old female child with cystic leukoencephalopathy without megalencephaly underwent successful CI, which indicated white matter and temporal lobe abnormalities should not deter pediatric CI [34]. Certainly, more studies are needed. In our study, few severe defects associated with speech auditory central system development and maturation were found on MR images of WML. In addition, many factors positively affect CI outcomes, including young age at implantation, HA, and hearing–speech rehabilitation experience [35,36]. Many participants in the study exhibited these characteristics, potentially explaining why they had satisfactory effects from CI. Further studies are needed to investigate in-depth short and longitudinal CI effects in children with WML temporal lobe involvement.

This study had several limitations. Firstly, it might have had a ceiling effect in the closed-set speech recognition tests, which limited the sensitivity of the association analysis between WML and speech recognition rates. Consequently, CAP scales were also completed to evaluate the auditory performance, which helped add to the reliability of results. Second, the sample size of our study is not large, which might affect the generalizability of the study. It was associated with the number of children with WMLs who received CI in our hospital. However, its participants were homogeneous, as all the participants were recruited based on strict inclusion criteria and exclusion criteria, which helped assess the reliability of the results. Third, the distribution of WMLs’ degrees was unbalanced, with scores of 1 and 2 accounting for about 70% of the WML group. It might be due to the comprehensive screening strategy for CI candidates with WMLs. It could be addressed by studies designed with a large sample size and a balanced distribution of the Fazekas score. Lastly, the WMLs’ degree was not evaluated post-CI. Based on the benefit–risk assessment, MRI scans were not performed post-CI. However, no participants reported any symptoms or stopped using their implant, indirectly indicating that their WMLs were stable during the first two years post-CI.

## 5. Conclusions

In conclusion, children with stable asymptomatic WMLs screened with PCES-WML obtained demonstrable benefit in auditory and speech–language abilities at 24 months post-CI. In addition, their speech recognition, auditory performance, and speech intelligibility were not different from CI peers with normal white matter during the first 24 months of implant use. Therefore, stable asymptomatic WML might not be associated with poor auditory and speech intelligibility post-CI, which indicates that it was feasible to use the comprehensive assessments, PCES-WML, to screen suitable candidates with WMLs who are likely to present with a good prognosis.

## Figures and Tables

**Table 1:** Fazekas scale.

(1) periventricular hyperintense (PVH)
0 = absence
1 = “caps” or pencil-thin lining
2 = smooth “halo”
3 = irregular periventricular signal extending into the deep white matter
(2) deep white matter hyperintense (DWMH)
0 = absence
1 = punctate foci
2 = beginning confluence
3 = large confluent areas

**Table 2:** Clinical characteristics of study participants.

Parameters	Total (*n* = 129)	WML Group (*n* = 43)	Control Group (*n* = 86)
Gender, *n* (%)			
Boys	73 (56.6)	24 (55.8)	49 (57.0)
Girls	56 (43.4)	19 (44.2)	37 (43.0)
Parental literacy, *n* (%)			
Senior middle school and above	49 (38.0)	15 (34.9)	34 (39.5)
Junior middle school and below	80 (62.0)	28 (65.1)	52 (60.5)
Residential setting, *n* (%)			
Urban	36 (27.9)	6 (14.0)	30 (34.9)
Rural	93 (72.1)	37 (86.0)	56 (65.1)
Residual hearing pre-CI, *n* (%)			
No (PTA: 0–120 dB nHL)	65 (50.4)	20 (46.5)	45 (52.3)
Yes	49 (49.6)	23 (53.5)	41 (47.7)
Experience of HA pre-CI, *n* (%)			
3–6 months	27 (20.9)	2 (4.7)	25 (29.1)
7–12 months	36 (27.9)	11 (25.6)	25 (29.1)
>12 months	66 (51.2)	30 (69.8)	36 (41.9)
Experience of rehabilitation pre-CI, *n* (%)			
>3 months rehabilitation from institute	90 (69.8)	36 (83.7)	54 (62.8)
Others	39 (30.2)	7 (16.3)	32 (37.2)
Implanted ear, *n* (%)			
Left	16 (12.4)	2 (4.7)	14 (16.3)
Right	113 (87.6)	41 (95.3)	72 (83.7)
Device, *n* (%)			
Nucleus	78 (60.5)	20 (46.5)	58 (67.4)
MED-EL	33 (25.6)	14 (32.6)	19 (22.1)
Advanced Bionics	18 (14.0)	9 (20.9)	9 (10.5)
Age at CI (years), Median (interquartile range)	3.5 (2.8, 5.4)	3.8 (3.0, 5.7)	3.4 (2.7, 5.0)
MMAIS/IT-MMAIS score pre-CI, Median (interquartile range)	8 (6, 10)	9 (6, 11)	8 (5, 10) ^a^

^a^ one missing MMAIS/IT-MMAIS score. Abbreviations: CI: cochlear implantation, HA: hearing aid, MAIS/IT-MAIS: Meaningful Auditory Integration Scale/Infant Toddler–Meaningful Auditory Integration Scale, PTA: pure tone audiometry, WML: white matter lesion.

**Table 3:** Distribution of Fazekas scores and involved areas of WML.

Parameters	WML Group (*n* = 43)
Fazekas score ^a^	
1	15 (34.9%)
2	15 (34.9%)
3	2 (4.7%)
4	5 (11.6%)
5	4 (9.3%)
6	2 (4.7%)
Involved Area	
Periventricular	25/43
Deep white matter	32/43
Frontal lobe	31/43
Parietal lobe	22/43
Occipital lobe	11/43
Temporal lobe	10/43

^a^ assessment on the second MRI scan before surgery. Abbreviations: MRI: magnetic resonance imaging, WML: white matter lesions.

**Table 4:** Auditory and speech outcomes at 1, 12 and 24 months post-CI.

	Variables	Mean (SD)/Median (25th, 75th Percentile)		
		1 Month	12 Months	24 Months
WML group	Mandarin Chinese speech recognition rates (%) ^a^			
	tone	23.5 (31.8)	73.4 (28.1)	84.8 (23.8)
	disyllable	21.7 (27.0)	77.7 (26.1)	87.9 (20.9)
	short sentence	13.2 (18.3)	75.2 (25.6)	85.8 (20.8)
	CAP ^b^	1, (1, 3)	4, (3, 5)	5, (4, 6)
	SIR ^b^	1, (1, 1)	3, (2, 3)	4, (3, 5)
control group	Mandarin Chinese speech recognition rates (%) ^a^			
	tone	26.5 (32.8)	72.5 (31.6)	89.0 (19.7)
	disyllable	26.9 (33.0)	76.7 (28.9)	89.7 (20.4)
	short sentence	21.7 (32.1)	75.1 (29.8)	88.0 (20.6)
	CAP ^b^	1, (1, 2)	4, (4, 5)	5, (4, 6)
	SIR ^b^	1, (1, 1)	3, (2, 3)	4, (3, 5)

^a^ mean (SD), ^b^ median (25th, 75th percentile). Abbreviations: CAP: category of auditory performance, CI: cochlear implantation, SIR: speech intelligibility rate, WML: white matter lesion.

**Table 5:** Sensitivity analysis of WML with CI outcomes.

Dependent Variables	β/OR (95% CI)
Tone recognition rate ^a^	−0.006 (−0.090 to 0.078, *p* = 0.885)
Disyllable recognition rate ^a^	−0.037 (−0.112 to 0.038, *p* = 0.332)
Short sentence recognition rate ^a^	−0.042 (−0.110 to 0.027, *p* = 0.235)
CAP score ≥ 4 ^b^	0.630 (0.300 to 1.324, *p* = 0.223)
SIR score ≥ 3 ^b^	0.652 (0.287 to 1.484, *p* = 0.308)

^a^ β (95% CI), ^b^ OR (95% CI). The results were based on model 3 (adjusted model) by restricting subjects to those who received CI at younger than 7 years of age (*n* = 117). Models were adjusted for time after activation, age at implantation, parental literacy, residual hearing, experience of rehabilitation pre-CI, and MMAIS/IT-MMAIS score pre-CI.

**Table 6:** Association of Fazekas score of WML and time after activation with CI outcomes.

Dependent Variable	Independent Variable	β/OR (95% CI)
Tone recognition rate ^a^	Fazekas score	0.0003 (−0.031 to 0.030, *p* = 0.983)
	Time 1	0 (referent)
	Time 2	0.500 (0.392 to 0.608, *p* < 0.001)
	Time 3	0.614 (0.501 to 0.727, *p* < 0.001)
Disyllable recognition rate ^a^	Fazekas score	0.007 (−0.020 to 0.035, *p* = 0.603)
	Time 1	0 (referent)
	Time 2	0.561 (0.465 to 0.657, *p* < 0.001)
	Time 3	0.663 (0.570 to 0.756, *p* < 0.001)
Short sentence recognition rate ^a^	Fazekas score	0.051 (−0.029 to 0.132, *p* = 0.213)
	Time 1	0 (referent)
	Time 2	0.641 (0.557 to 0.725, *p* < 0.001)
	Time 3	0.747 (0.678 to 0.816, *p* < 0.001)
CAP score ≥ 4 ^b^	Fazekas score	0.979 (0.937 to 1.022, *p* = 0.326)
	Time 1	1 (referent)
	Time 2	1.834 (1.583 to 2.125, *p* < 0.001)
	Time 3	2.209 (1.951 to 2.501, *p* < 0.001)
SIR score ≥ 3 ^b^	Fazekas score	0.994 (0.953 to 1.038, *p* = 0.800)
	Time 1	1 (referent)
	Time 2	1.750 (1.508 to 2.032, *p* < 0.001)
	Time 3	2.424 (2.197 to 2.674, *p* < 0.001)

^a^ β (95% CI), ^b^ OR (95% CI). Independent variables included time after activation, age at implantation, Fazekas score, parental literacy, residual hearing, experience of rehabilitation pre-CI, and MMAIS/IT-MMAIS score pre-CI.

**Table 7:** Spearman rank correlations between Fazekas scores and CI outcomes in the WML group.

		Mandarin Chinese Speech Recognition			CAP	SIR
		Tone	Disyllable	Short Sentence		
12 months post-CI	Correlation coefficient (*r_s_*)	−0.185	0.125	0.165	−0.137	0.057
	*p* value	0.241	0.423	0.291	0.382	0.718
24 months post-CI	Correlation coefficient (*r_s_*)	−0.028	0.015	0.082	−0.025	0.026
	*p* value	0.860	0.924	0.601	0.876	0.870

Abbreviations: CAP: category of auditory performance, CI: cochlear implantation, SIR: speech intelligibility rate, WML: white matter lesion.

## Data Availability

Data are unavailable due to privacy and ethical restrictions.

## Supplementary Materials

The following supporting information can be downloaded at https://www.mdpi.com/article/10.3390/brainsci13111540/s1, Table S1: Categories of auditory performance (CAPs) criteria; Table S2: Speech intelligibility rating (SIR) criteria; Table S3: Outcomes at 1, 12, and 24 months post-CI in cases with Fazekas scores of 6 (cases 12, 42) and 5 (cases 5, 8, 15, 36); Table S4: Outcomes at 24 months post-CI in children with and without temporal lobe involvement.
