# Supplementary material for: Developmental Auditory and Speech–Language Performance in Pediatric Cochlear Implantation Recipients with Stable White Matter Lesions
Source: Brain Sci. 2023 Nov 1;13(11):1540. doi: 10.3390/brainsci13111540 (PMC10669646; doi:10.3390/brainsci13111540)
Supplement: Supplementary file 1 [file brainsci-13-01540-s001.zip › brainsci-2647616-supplementary.pdf]

Supplemental Table S1. Categories of auditory performance (CAPs) criteria

Criteria	Category
No awareness of environmental sounds	0
Awareness of environmental sounds	1
Response to speech sounds	2
Identification of environmental sounds	3
Discrimination of some speech sounds without lip-reading	4
Understanding of common phrases without lip-reading	5
Understanding of conversation without lip-reading	6
Use of telephone with known listener	7

Supplemental Table S2. Speech intelligibility rating (SIR) criteria

Criteria	Category
Connected speech is unintelligible. Pre-recognisable words in spoken language, primary mode of communication may be manual	1
Connected speech is unintelligible. Intelligible speech develops in single words when contextual and lip-reading cues are available	2
Connected speech is intelligible to a listener who concentrates and lip-reads	3
Connected speech is intelligible to a listener who has a little experience of a deaf person's speech	4
Connected speech is intelligible to all listeners. Child is understood easily in everyday contexts	5

Supplemental Table S3. Outcomes at 1, 12, and 24 months post-CI in cases with Fazekas scores of 6 (case-12, 42) and 5 (case-5, 8, 15, 36)

Case Number	age at CI (y)	months post-CI	Mandarin Chinese speech recognition(%)			CAP	SIR
			tone	disyllable	short sentence		
case-12[a]	3.8	1	0	0	0	2	1
		12	75	100	95	4	3
		24	100	100	100	7	5
case-42	3.1	6	45	43	45	2	1
		12	60	47	45	3	1
		24	80	66	80	4	2
case-5[b]	6.8	1	40	43	5	1	1
		12	50	87	70	3	3
		24	55	80	85	3	3
case-8[c]	5.7	1	55	65	65	2	1
		12	98	100	95	2	1
		24	100	100	100	2	2
case-15	3.4	1	0	7	0	3	2
		12	100	100	100	4	3
		24	100	100	100	4	4
case-36[d]	2.9	1	10	0	0	1	1
		12	80	83	75	4	2
		24	100	100	95	5	4

[a] the MRI is showed in Figure 1(B), [b] the MRI is showed in Figure 3(D), [c] the MRI is showed in Figure 2(C), [d] the MRI is showed in Figure 2(D)

Abbreviations: CAP: category of auditory performance, CI: cochlear implantation, SIR: speech intelligibility rate

Supplemental Table S4. Outcomes at 24 months post-CI in children with and without temporal lobe involvement

Variable		temporal lobe involvement		p^a
		positive(n=10)	negative(n=33)	
speech	tone	92 (78, 100)	100 (74, 100)	0.738
recognition [b]	disyllable	95 (80, 100)	100 (85, 100)	0.640
(%)	short sentence	83 (79, 96)	100 (75, 100)	0.286
CAP [b]		5, (4, 5)	5, (4, 6)	0.402
SIR [b]		4, (3, 5)	4, (3, 5)	0.921

[a] By Mann Whitney U test, [b] Median (25th, 75th percentile)

Abbreviations: CAP: category of auditory performance, CI: cochlear implantation, SIR: speech intelligibility rate.
